# The effect of metformin treatment during primary influenza infection on heterologous challenge in young and aged mice

**DOI:** 10.3389/fragi.2026.1736105

**Published:** 2026-06-17

**Authors:** Dominique E. Teskey, Andreia N. Cadar, Nagaraju Marka, Zena L. Haddad, Darlene A. Djaba, Blake L. Torrance, Ferris El-Tayyeb, Bishajit Sarkar, Aadrita Hazra, Jenna M. Bartley

**Affiliations:** 1 UConn Center on Aging, University of Connecticut School of Medicine, Farmington, CT, United States; 2 Department of Immunology, University of Connecticut School of Medicine, Farmington, CT, United States

**Keywords:** aging, geroscience, influenza, metformin, T cell memory

## Abstract

**Introduction:**

Respiratory illnesses like influenza and SARS-CoV-2 disproportionately affect older adults, leading to severe complications and high mortality rates. Age-related immune dysregulation impairs infection responses and hinders recovery. The geroscience hypothesis suggests that targeting biological aging can enhance overall healthspan. Mitochondrial dysfunction and dysregulated nutrient sensing, hallmarks of aging, profoundly affect metabolism and cellular function. Metformin, an FDA-approved diabetes drug, is a candidate anti-aging drug and has been shown to positively impact immune cell function in many contexts. However, the totality of these effects on immune cells remains under investigation. Here, we aim to determine if metformin treatment could improve immune memory responses by utilizing a heterologous flu challenge model.

**Methods:**

Young and aged mice were given control or metformin treated chow for 6 weeks prior to being infected with a sublethal dose of H3N2 influenza virus A/HKx31 (X31). Control and treated chow continued until 10 days post infection to examine the effects of metformin on immune memory formation. Mice were then allowed to recover and at 30 days post initial infection and were challenged with a heterologous H1N1 influenza virus A/Puerto Rico/8/34 (PR8). Mice were sacrificed on day 0 (prior to secondary flu challenge), and at 5, 7, 10, and 14 days post-secondary infection to unveil changes in the kinetics of immune responses.

**Results:**

Metformin altered only some aspects of immune responses during secondary flu challenge, and more so in young mice compared to aged mice. More specifically, we did not observe improved T cell memory populations in the lungs following primary flu infection in aged metformin treated mice compared to aged control treated mice. Moreover, while aged metformin treated mice had modestly improved weight loss during heterologous challenge, they had transiently increased lung viral load compared to aged control treated mice.

**Discussion:**

This suggests that metformin could not overcome the totality of aging to improve T cell memory responses. Thus, while metformin has been shown to have many benefits in a variety of aging conditions, its specific utility in improving age-related declines in immune memory formation during infection is unclear in our studies. More research is necessary to determine how metformin can target aging physiology and T cell function to enhance immune responses, and importantly, understand the limitations of its utility in aging populations.

## Introduction

A prominent consequence of aging is diminished immune function, leading to decreased ability to fight infections. While respiratory illnesses like influenza (flu), and more recently, SARS-CoV-2 affect individuals of all ages, older adults face a disproportionately high risk for severe disease, complications, and death (Nikolich-Zugich, 2018; Mueller et al., 2020). For example, 67% of flu-related deaths occurred in individuals aged 65 and older worldwide (Paget et al., 2019). Beyond this, older adults also face an elevated risk of hospitalization and complications post infection, including secondary pneumonia, impaired cognitive function, heightened cardiovascular events, increased fracture or fall risk, and difficulties in performing daily activities (Mueller et al., 2020; Czaja et al., 2019; Axelsson et al., 2022).

Age-related immune declines not only impact the ability to fight active infections but also hinder the recovery process and alter immune memory formation (Nikolich-Zugich, 2018; Weyand et al., 2016). Older adults exhibit impaired cell-mediated and humoral immune responses, characterized by diminished T cell memory formation and reduced antibody quantity and quality (Nikolich-Zugich, 2018; Weyand et al., 2016; Mittelbrunn and Kroemer, 2021). Thus, older adults are not well-protected from a secondary infection. Similarly, older adults have reduced flu vaccination responses which also leaves them susceptible to infection (Gustafson et al., 2020; Sadighi Akha, , 2018). Overall, impaired memory formation with aging remains a major issue in older populations leaving them vulnerable to severe infections despite prior infection or vaccination. Consequently, identifying strategies to enhance infection resistance and vaccination responses in older adults becomes a critical public health imperative (Nikolich-Zugich, 2018; Weyand et al., 2016).

Recent geroscience-focused research accentuates the potential of targeting biological aging as a promising strategy to ameliorate age-related declines in physiology and resilience, ultimately enhancing overall healthspan (Lopez-Otin et al., 2013; Lopez-Otin et al., 2023). While targeting specific deficits in aged immune system is under investigation, this approach may be insufficient due to the complex and coordinated nature of immune responses to viral infection. Thus, targeting the hallmarks of aging presents a broader approach to enhance responses to viral challenge and promote the generation of immunological memory. Two prominent hallmarks of aging, mitochondrial dysfunction and dysregulated nutrient sensing exert profound effects on metabolic function at both systemic and cellular levels (Lopez-Otin et al., 2013; Lopez-Otin et al., 2023). Mammalian target of rapamycin (mTOR) and AMP-activated protein kinase (AMPK), recognized as master regulators of metabolism, are altered with aging in multiple cell types and tissues. Importantly, proper immune cell function is intricately tied to cellular metabolism, with modulation of the mTOR/AMPK pathways being pivotal for T cell activation, T helper (Th) subset differentiation, effector function, and memory cell generation (Chi, 2012; Sun et al., 2016). More specifically, the formation of memory T cells is dependent AMPK activation and enhancement of fatty acid oxidation (Pearce et al., 2009). Taken together, targeting mitochondrial dysfunction and dysregulated nutrient sensing may provide an important avenue for addressing age-related immune declines.

Drugs modulating AMPK/mTOR pathways, such as metformin and rapamycin, have garnered interest for combating various age-related diseases (Justice et al., 2021; Selvarani et al., 2020). Metformin, an FDA-approved diabetes drug, stands out as a candidate that targets many hallmarks of aging, making it an ideal contender for addressing aging biology comprehensively (Justice et al., 2021). Importantly, metformin activates AMPK to increase fatty acid oxidation and has profound impact on metabolic pathways. Beyond its metabolic targets, metformin has exhibited positive effects on various immune cell types, both through direct immunometabolic modulation and by alleviating chronic pro-inflammatory signaling known to dysregulate immune responses (Justice et al., 2021).

Importantly, metformin has been shown to promote CD8 T cell memory formation in young mice through AMPK activation and fatty acid oxidation (Pearce et al., 2009), but this has yet not been replicated in aged mice. Further evidence has demonstrated that metformin can target the immune system by impacting metabolism. More specifically, metformin was found to ameliorate thymus degeneration of mice by regulating mitochondrial function (Yang et al., 2022). Additionally, *in vitro* metformin treatment has been shown to improve mitochondrial function, enhance autophagy, and mitigate age-related Th17-driven inflammation in CD4 T cells from older adults (Sun et al., 2016; Bharath et al., 2020; Duan et al., 2019).

Increasing evidence demonstrates metformin’s immunoprotective benefits and ability to improve immune responses. In diabetic older adults, metformin treatment leads to increased B cell function and to improved antibody responses to flu vaccine compared to other oral hypoglycemics (Frasca et al., 2021; Diaz et al., 2017). Further, in a model of acute *Streptococcus pneumoniae*, metformin was shown to attenuate inflammatory responses and enhance antibody production (Lee et al., 2022). Additionally, we have previously shown that metformin treatment in nondiabetic older adults increases circulating T follicular helper (Tfh) cells following flu vaccination and improves aspects of CD4 T cell exhaustion (Martin et al., 2023a). Tfh cells are a specialized subset of CD4^+^ T cells that are essential for providing help to follicular B cells within germinal centers in secondary lymphoid tissues (Gustafson et al., 2018). They are an essential component of the adaptive immune response. Overall, while metformin has been shown to improve certain aspects of immune responses in young mice and in individuals with diabetes, its effects on the broad immune deficits observed with aging remain largely underexplored.

Targeting the physiology of aging and T cell function with metformin emerges as a novel avenue to alleviate systemic inflammation, mitigate immune cell exhaustion, and enhance overall immune responses, ultimately leading to improved immune protection in older adults. To better understand how metformin can modulate memory responses with aging, we utilized a heterologous flu challenge model in young and aged mice. To isolate the impact of metformin on memory formation and function, mice were treated with metformin prior to and up to 10 days post primary flu infection. We hypothesized that metformin treatment would increase T cell memory formation in both young and aged mice and improve protection from heterologous flu challenge. Our findings reveal that metformin improved only certain aspects of immune responses during heterologous flu challenge and most changes in immune responses occurred in young mice. In fact, transient negative effects were observed in metformin treated aged mice. Thus, we postulate that metabolic skewing may have impaired effector responses, or that metformin did not provide sufficient stimulus to overcome the numerous immune deficits associated with aging and enhance memory T cell formation, thereby limiting the benefits against secondary flu challenge.

## Materials and methods

### Mice

Young (3–4 months) and aged (18–19 months) C57BL/6JN male mice were purchased from Jackson Laboratories or generously provided by the National Institute on Aging Rodent Colony and housed at UConn Health. Mice underwent a 2-week acclimation period prior to any experimental procedures and were grouped by weight matching. All mice underwent examinations at the time of sacrifice by CO_2_ asphyxiation and animals with gross pathology (e.g., visible tumors) were excluded from analysis. All mice were housed in a climate-controlled environment and were provided chow (details below) and water *ad libitum*. All mice were cared for in accordance with the recommendations in the Guide for the Care and Use of Laboratory Animals of the National Institutes of Health. All procedures were approved by the UConn Health Institutional Animal Care and Use Committee (IACUC).

### Mouse chow (standard and metformin)

Mice were given control or metformin treated chow *ad libitum* for 6 weeks prior to primary infection which continued for an additional 10 days after primary flu infection. Control (standard) chow consisted of 18 kcal% fat, 58 kcal% carbohydrate and 24 kcal% protein (Teklad global 18% protein rodent diet, ENVIGO, Indianapolis, IN). Treated diets were ceased 10 days after primary infection to assess the effects of metformin on immune memory formation. Metformin was formulated in the chow at 150 mg/kg (0.1%) food (150 ppm, or about 20 mg/mouse/day for a 30 g mouse, Supplementary Table S1) (Martin-Montalvo et al., 2013).

### Viral infection

For primary viral infection, mice were anesthetized using 3%–4% isoflurane, intranasally infected with a sublethal dose of a H3N2 influenza virus A/HKx31 (x31, 3000 EID50). Thirty days later, mice were anesthetized using 3%–4% isoflurane and intranasally infected with a H1N1 influenza virus A/Puerto Rico/8/34 (PR8, 500 EID50). Mice were monitored and weighed daily throughout both infections to assess overall health and percent weight loss as an indication of infection progress. One control and one metformin treated aged mice were humanely euthanized and excluded from analysis due to losing beyond 30% of their original body weight during primary infection.

### Bronchoalveolar lavage (BAL) collection and albumin and cytokine quantification

Bronchoalveolar lavage (BAL) was collected by flushing lungs once with 1 mL of PBS at time of sacrifice. After collection, BAL was centrifuged (8,500 rpm for 15 min at 4 °C) to remove cells and debris and supernatant was frozen at −80 °C until analyses. Concentration of albumin in BAL was determined using an albumin ELISA kit (Abcam) following the manufacturer’s protocol. Cytokine analysis was performed using a magnet-based multiplex ELISA (Millipore) and analyzed using a MAGPIX plate reader (Luminex).

### Lung viral load

Following sacrifice and BAL collection, lungs were flash frozen in liquid nitrogen. Lung tissue was homogenized using a handheld homogenizer (Pro Scientific) and RNA was isolated via standard trizol/chloroform (Invitrogen Life Technologies and Sigma Aldrich, respectively) extraction per the manufacturer’s protocol. cDNA was synthesized using iScript cDNA synthesis kit (Bio-Rad) using the manufacturers protocol. Viral load was determined by RT-qPCR for PR8 acid polymerase (PA) gene compared to a standard curve of known PA copy numbers as previously published (Jelley-Gibbs et al., 2007; Keilich et al., 2020). The following primer and probe were used: forward primer, 5′-CGG​TCC​AAA​TTC​CTG​CTG​A-3′; reverse primer, 5′- CAT​TGG​GTT​CCT​TCC​ATC​CA-3′; probe, 5′-6-FAM-CCAAGTCATGAAGGAGAGGGAATACCGCT-3′ (Integrated DNA Technologies).

### Antibody quantification

Serum was obtained from whole blood collected via cardiac puncture upon sacrifice by CO_2_ asphyxiation. Bronchoalveolar lavage (BAL) was collected as described above. Samples were serially diluted and analyzed for whole X31, whole PR8, or NP IgG levels via ELISA as previously described (Lefebvre et al., 2016; Torrance et al., 2023).

### Tissue processing and flow cytometry

Following sacrifice by CO_2_ asphyxiation, lungs were mechanically and enzymatically digested (100 U/mL collagenase, Gibco). For flow cytometry, cells were incubated with Fc block (anti-CD16/32, ThermoFisher) followed by staining with MHC Class I (H2-Db ASNENMETM) or MHC Class II (I-Ab QVYSLIRPNENPAHK) tetramers (generated by the NIH Tetramer Core Facility). Cells were subsequently stained with surface antibodies. Samples with only surface staining were fixed using 1% paraformaldehyde. Samples requiring intracellular staining were fixed and permeabilized using a FoxP3/Transcription Factor staining kit (ThermoFisher) prior to incubation with intracellular antibodies. The surface and intracellular antibodies used are listed in Supplementary Table S2. A Becton Dickinson (BD) LSR II cytometer was used, and analysis was performed using FlowJo software (BD).

### Statistics

All data are presented as mean ± standard error of the mean (SEM). Analyses were performed using Prism software (GraphPad). Statistical significance was calculated by two-way ANOVA (age x treatment) with Šídák’s posthoc corrections for multiple comparisons, and p-values <0.05 were considered significant. Comparisons between metformin and control groups were nonsignificant unless otherwise indicated.

## Results

### Effect of metformin on weight loss, antibody and T cell responses during primary flu infection

**FIGURE 1:**
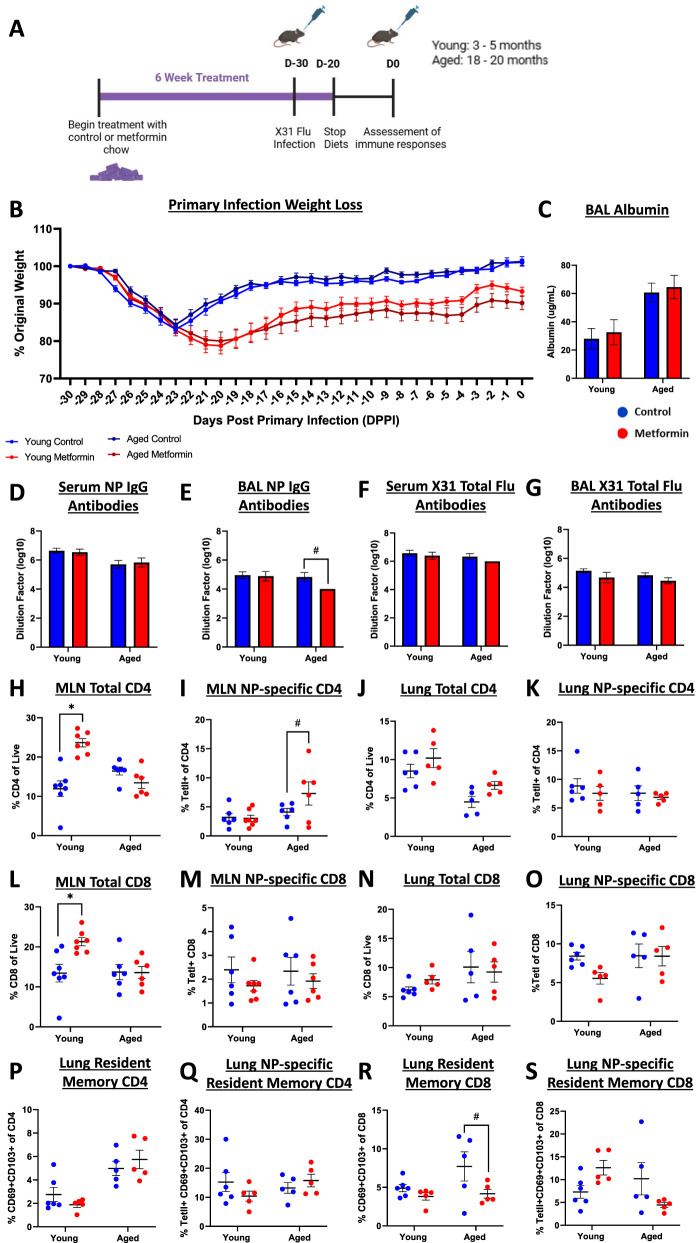
Experimental Design, weight loss and assessment of immune responses 30 days post primary A/HKx31 (X31) flu infection. Young and aged mice were fed control or metformin treated chow (150 mg metformin/kg chow) for 6 weeks prior to primary infection with H3N2 influenza virus A/HKx31 (X31). Diets were stopped 10 days post X31 flu infection to evaluate the effects of metformin on memory formation. 30 days post X31 flu infection, mice were sacrificed and the serum from cardiac puncture, bronchoalveolar lavage (BAL) fluid, the mediastinal lymph node (MLN), and lungs were analyzed. **(A)** Experimental Design. **(B)** Mice were weighed daily for 30 days following primary infection with X31 and percent body mass change was calculated. **(C)** BAL Albumin was assessed to measure lung damage. NP IgG Antibody levels were assessed in the serum **(D)** and BAL **(E)**. X31 whole virus IgG levels were measured in the serum **(F)** and BAL **(G)**. Total **(H)** and MHC Class II Tetramer NP + **(I)** CD4 T cell frequencies were assessed via flow cytometry in the MLN. Total **(J)** and MHC Class II Tetramer NP + **(K)** CD4 T cell frequencies were assessed via flow cytometry in the lungs. Total **(L)** and MHC Class I Tetramer NP + **(M)** CD8 T cell frequencies were assessed in the MLN. Total **(N)** and MHC Class I Tetramer NP + **(O)** CD8 T cell frequencies were assessed in the lungs. Total **(P)** and MHC Class II Tetramer NP + **(Q)** resident memory (CD69+CD103+) CD4 T cell frequencies were assessed in the lungs. Total **(R)** and MHC Class I Tetramer NP + **(S)** resident memory (CD69+CD103+) CD8 T cell frequencies were assessed in the lungs. Statistical significance was calculated by two-way ANOVA with Šídák’s posthoc corrections for pairwise comparisons of control vs. metformin-treated mice. Significance was set at p ≤ 0.05, indicated by *. Trends (0.05 < p ≤ 0.1) indicated by #. **(B)**: n = 10 --14/group , **(C)**: n = 5 /group , **(D–G)**: n = 5 --7/group, **(H–S)**: n = 5 --7/group.

We, and others, have shown significant age-related differences in response to flu infection in mice, including increased weight loss, slower viral clearance, and increased albumin in the bronchoalveolar lavage (BAL) in aged mice (Lefebvre et al., 2016; Sun et al., 2019; Bh et al., 2015; Bartley et al., 2016; Toapanta and Ross, 2009). Similarly, we and others have shown deficits in memory formation and protection during heterologous flu challenge during aging (Paquette et al., 2014; Maue et al., 2009; Salam et al., 2013). Thus, we sought to determine if metformin treatment could improve functional T cell memory responses during a heterologous flu challenge. We hypothesized that metformin treatment prior to heterologous flu challenge in aged mice would improve T cell memory formation and humoral responses, as this drug has previously been shown to enhance memory CD8 T cell responses in young mice (Pearce et al., 2009) and improve antibody responses in diabetic older adults (Frasca et al., 2021; Diaz et al., 2017). We first treated young (3–5 month old) and aged (18–20 month old) male mice with 0.1% metformin chow for 6 weeks. Following 6 weeks of treatment, mice were infected with a sublethal dose of a H3N2 influenza virus A/HKx31 (X31, primary flu infection) (Figure 1A). Metformin diets were stopped 10 days post-primary X31 infection (DPPI) to isolate the effects of metformin on immune memory formation and function. During primary infection, mice were weighed daily for 30 days to assess infection progression (Figure 1B). As our group has previously reported (Bartley et al., 2016), aged mice experience more severe and prolonged weight loss compared to young mice up to 30 DPPI (Figures 1B,C, main age effect p < 0.001). Metformin treatment did not impact flu-induced weight loss in young or aged throughout primary infection (Figure 1B).

Next, we assessed local and systemic responses to primary X31 infection at 30 DPPI. To assess lung damage (Bh et al., 2015), we analyzed albumin concentrations in the bronchoalveolar lavage fluid (BAL). Here, we found that aged mice exhibited increased albumin concentrations compared with young mice (age effect: p < 0.001), regardless of treatment (Figure 1C). Next, we evaluated flu nucleoprotein (NP) IgG and whole virus (X31) IgG antibodies in bronchoalveolar lavage (BAL) fluid and serum. We first examined flu nucleoprotein (NP) specific antibody responses (Figures 1D,E), as antibodies against this conserved internal protein provide cross-reactive protection to other Influenza A strains. Although no differences in serum NP IgG antibodies were observed between young and aged or control and metformin treated mice, we observed a trending decrease (p = 0.08) in BAL NP IgG antibody titers in aged metformin treated mice compared to aged control mice (Figure 1E). No changes in whole flu (X31) IgG antibodies were observed between young and aged mice or with metformin treatment in the serum or BAL (Figures 1F,G).

We continued by evaluating changes to T cell responses with metformin treatment at 30 DPPI. We assessed total CD4 T cell, NP-specific CD4 T cell, total CD8 T cell and NP-specific CD8 T cell responses in the mediastinal lymph node (MLN) and lungs. Young, but not aged, metformin treated mice had increased frequency of total CD4 and CD8 T cells in the MLN (Figures 1H,L), but not NP-specific CD4 or NP-specific CD8 T cell frequency (Figures 1I,M). Aged metformin treated mice only showed a trending increase in NP-specific CD4 T cells in the MLN compared to control treated aged mice (Figures 1J,K; Figures 1N,O). No changes in cell number of CD4 or CD8 T cells were observed in the MLN or lungs in any group except for a significant decrease in NP-specific CD8 T cells in the MLN of young metformin treated mice (Supplementary Figure S2).

We next assessed tissue resident memory T cell (CD69^+^CD103+) populations in the lungs at 30 DPPI to determine the effects of metformin on T cell memory formation after primary infection. Metformin treatment did not impact the generation of total or NP-specific resident memory CD4 T cells in the lungs of young or aged mice at 30 DPPI (Figures 1P,Q). Interestingly, we found that metformin treated aged mice had a trending decrease in the frequency of total (p = 0.0515), but not NP-specific lung resident memory CD8 T cells (Figures 1R,S). Further, both groups recovered similarly in terms of lung damage and inflammation at this point (Supplementary Figure S1). While these findings suggest that metformin has minimal effects on the magnitude of total, NP-specific and memory CD4 and CD8 T cell populations at 30DPPI in aged mice, it is possible that metformin impacted other aspects of immune responses. Thus, we assessed the functionality and effectiveness of the immunological protection generated from primary flu infection against a secondary heterologous flu challenge.

### Effect of metformin treatment on early immune responses following secondary flu challenge

**FIGURE 2:**
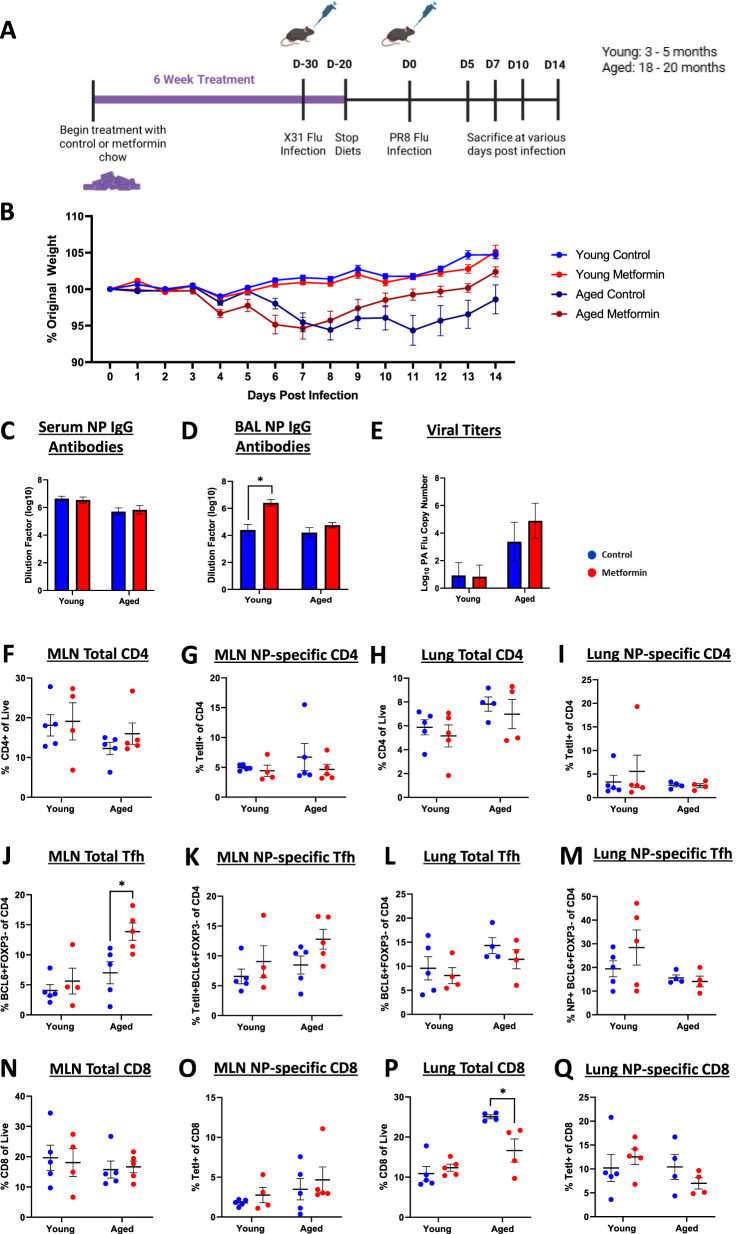
Assessment of immune responses at 5 days post secondary A/Puerto Rico/8/34 (PR8) flu infection. Young and aged, control or metformin treated mice were initially infected with H3N2 influenza virus A/HKx31 (X31), then challenged 30 days later with H1N1 influenza virus A/Puerto Rico/8/34 (PR8) and sacrificed at 5 days post secondary infection. At the time of sacrifice, serum from cardiac puncture, bronchoalveolar lavage (BAL) fluid, the mediastinal lymph node (MLN), and lungs were analyzed. **(A)** Experimental design. **(B)** Mice were weighed daily to assess percent original weight lost during secondary flu challenge. Flu nucleoprotein (NP) IgG antibody levels in the serum **(C)** and BAL **(D)** were assessed. **(E)** Lung viral load was assessed at 5 days post secondary infection. Total **(F)** and MHC Class II Tetramer NP + **(G)** CD4 T cell frequencies were evaluated in the MLN. Total **(H)** and MHC Class II Tetramer NP + **(I)** CD4 T cell frequencies were evaluated in the lungs. Total **(J)** and MHC Class II Tetramer NP + **(K)** T follicular helper T cell (Tfh; CD4+, BCL6+FOXP3‐) frequencies were assessed in the MLN. Total **(L)** and MHC Class II Tetramer NP + **(M)** Tfh cells frequencies were assessed in the lungs. Total **(N)** and MHC Class I Tetramer NP + **(O)** CD8 T cell frequencies were assessed in the MLN. Total **(P)** and MHC Class I Tetramer NP + **(Q)** CD8 T cell frequencies were assessed in the lungs. Statistical significance was calculated by two-way ANOVA with Šídák’s posthoc corrections for pairwise comparisons of control vs. metformin-treated mice. Significance was set at p ≤ 0.05, indicated by *. Trends (0.05 ≤ p ≤ 0.1) indicated by #. 2B: n = 31 --33/group from combined experiments, **(C,D)**: n = 5/group, **(E)**: n = 6 --7 /group, **(F–Q)**: 4 --5/group.

To assess the effect of metformin on the generation of functional memory responses, 30 days following primary X31 infection (day 0), mice were infected with a sublethal heterologous H1N1 influenza virus A/Puerto Rico/8/34 (PR8) and monitored throughout secondary infection (Figure 2A). Overall, young mice did not lose weight during the secondary challenge, regardless of whether they received metformin or control treatment, indicating robust and comparable immunological protection against heterologous flu challenge (Figure 2B). Aged mice experienced more severe and prolonged weight loss compared to young mice during secondary infection. However, aged mice experienced less overall weight loss during secondary infection compared to primary infection (Figures 1B, 2B). Aged metformin treated mice experienced significantly more weight loss than aged control mice at 6 days post-secondary infection (DPSI), however recovered more weight compared to control aged mice from 10–14 DPSI. Importantly, while statistically significant, the percentage weight change during heterologous flu challenge was minimal, making its translational relevance unclear.

To further evaluate early immune responses to secondary flu challenge, we examined antibody quantities, viral load, and T cell responses in the MLN and lungs. Young mice had increased BAL and serum NP IgG antibodies compared to aged mice (main age effect: p < 0.01 for both). In young mice only, metformin treatment increased BAL, but not serum, NP IgG antibodies at 5 DPSI compared to young control mice (Figures 2C,D). This suggests that metformin treatment may improve local antibody recall responses early during secondary challenge in young mice but not aged. Furthermore, we did not observe any differences in viral load between control and metformin young or aged mice (Figure 2E). However, overall aged mice had increased levels of virus in the lungs compared to young mice (main age effect: p < 0.05). Previous studies have shown that aged mice have slower viral clearance with aging (Lefebvre et al., 2016), which parallels our findings with heterologous challenge.

To assess early T cell memory responses, we identified total and NP-specific CD4 and NP-specific CD8 T cells in the MLN and lungs. Here, we observed that metformin did not influence total CD4 or NP-specific CD4 T cell frequency in the MLN or lung at 5 DPSI (Figures 2F–I). However, we saw a skewing towards T follicular helper cells (Tfh) in the MLN, but not the lungs at 5 DPSI in aged metformin treated mice compared to aged control treated mice (Figures 2J–M). Further, although no changes in total and NP-specific CD8 T cell frequency was observed in the MLN, metformin treatment led to a decrease in total, but not NP-Specific CD8 T cell frequency in the lungs of aged metformin treated mice only (Figures 2N–Q). Interestingly, we also observed a decrease in the number of total and flu-specific CD8 T cells in the lungs of aged metformin mice at 5 DPSI (Supplementary Figure S3). To determine the effects of the changes observed at 5 DPSI on overall flu outcomes, we continued to investigate later timepoints in secondary infection.

### Metformin treatment impacts lung inflammation and viral load at 7 DPSI in aged mice

**FIGURE 3:**
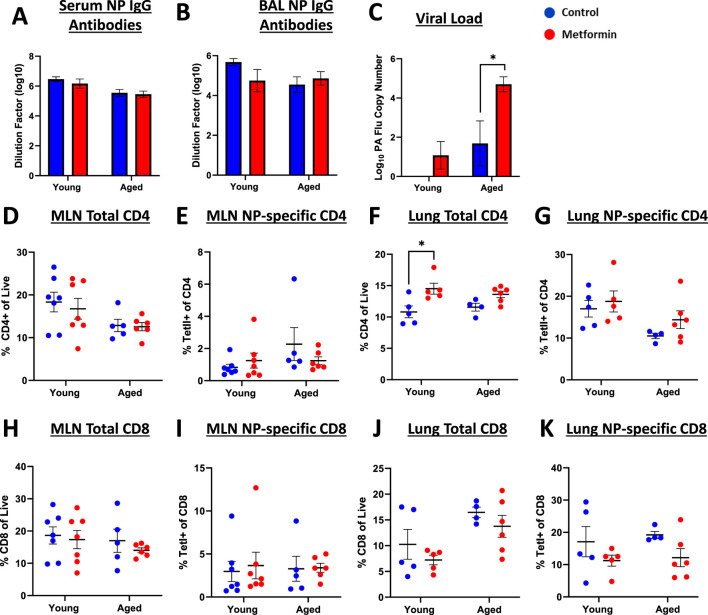
Assessment of immune responses at 7 days post secondary A/Puerto Rico/8/34 (PR8) flu infection. Young and aged, control or metformin treated mice were initially infected with H3N2 influenza virus A/HKx31 (X31), then challenged 30 days later with H1N1 influenza virus A/Puerto Rico/8/34 (PR8) and sacrificed at 7 days post secondary infection. At the time of sacrifice, serum from cardiac puncture, bronchoalveolar lavage (BAL) fluid, the mediastinal lymph node (MLN), and lungs were analyzed. **(A,B)** Flu nucleoprotein (NP) IgG antibody levels were analyzed in the serum **(A)** and BAL **(B)**. **(C)** Lung viral load was analyzed at 7 days post secondary infection. Total **(D)** and MHC Class II Tetramer NP + **(E)** CD4 T cell frequencies were evaluated in the MLN. Total **(F)** and MHC Class II Tetramer NP + **(G)** CD4 T cell frequencies were evaluated in the lungs. Total **(H)** and MHC Class I Tetramer NP + **(I)** CD8 T cell frequencies were evaluated in the MLN. Total **(J)** and MHC Class I Tetramer NP + **(K)** CD8 T cell frequencies were evaluated in the lungs. Statistical significance was calculated by two-way ANOVA with Šídák’s posthoc corrections for pairwise comparisons of control vs. metformin-treated mice. Significance was set at p ≤ 0.05, indicated by *. Trends (0.05 < p ≤ 0.1) indicated by #. **(A,B)**: n = 5 --7/group, **(C)**: n = 5 - - 7/group, **(D -K)**: n = 4 - - 7/group.

Mice were sacrificed at 7 DPSI for antibody titers, viral load and T cell response analyses to further evaluate the effects of metformin on the kinetics of a heterologous flu challenge. Metformin treatment did not influence NP IgG levels in the serum or BAL in young or aged mice at 7 DPSI (Figures 3A,B). Young mice overall had increased serum NP IgG compared to aged (main age effect: p < 0.01). Lung viral load was significantly increased in metformin treated aged mice compared to control (Figure 3C) and aged mice have increased viral load compared to young at 7 DPSI (main age effect: p < 0.001). This was accompanied by increased inflammation in the BAL at this time point. Aged metformin treated mice had significantly higher levels of classical inflammatory cytokines, e.g., G-CSF, IL1b, TNFa and IL-6, as well as Type 2 cytokines, e.g., IL-4, and IL‐10, in the BAL (Supplementary Figure S4). Young mice, conversely, had low levels of these cytokines indicating they were protected from severe secondary flu-induced lung inflammation. Surprisingly, despite these striking differences in lung viral load and inflammation, no significant differences were observed in total of NP-specific CD4 or CD8 T cell frequency or cell numbers in the MLN or lungs at 7 DPSI in aged mice (Figures 3D–K; Supplementary Figure S5). Interestingly, we observed an increase in frequency of total CD4 T cells in the lung of metformin treated young mice. It is unclear the meaning of this change given there are no alterations in NP-specific CD4 T cells in the lungs (Figures 3F,G).

### Metformin treatment promotes shift towards T follicular helper (Tfh) subsets in aged mice at 10 DPSI

**FIGURE 4:**
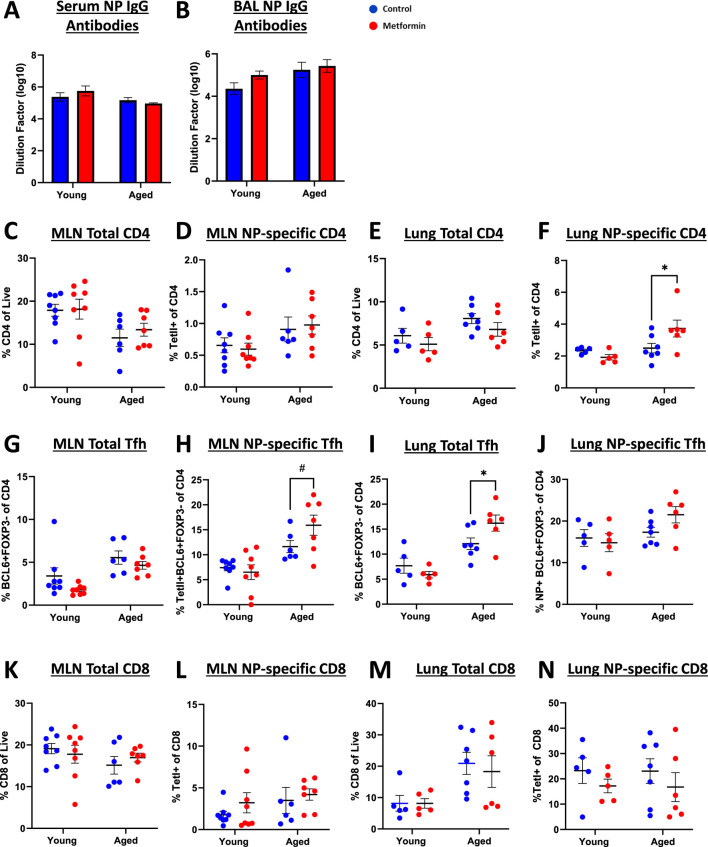
Assessment of immune responses at 10 days post secondary A/Puerto Rico/8/34 (PR8) flu infection. Young and aged, control or metformin treated mice were initially infected with H3N2 influenza virus A/HKx31 (X31), then challenged 30 days later with H1N1 influenza virus A/Puerto Rico/8/34 (PR8) and sacrificed at 10 days post secondary infection. At the time of sacrifice, serum from cardiac puncture, bronchoalveolar lavage (BAL) fluid, the mediastinal lymph node (MLN), and lungs were analyzed. Flu nucleoprotein (NP) IgG antibody levels were analyzed in the serum **(A)** and BAL **(B)**. Total **(C)** and MHC Class II Tetramer NP + **(D)** CD4 T cell frequencies were assessed in the MLN. Total **(E)** and MHC Class II Tetramer NP + **(F)** CD4 T cell frequencies were assessed in the lungs. Total **(G)** and MHC Class II Tetramer NP + **(H)** T follicular helper T cell (Tfh; CD4+, BCL6+FOXP3‐) frequencies were assessed in the MLN. Total **(I)** and MHC Class II Tetramer NP + **(J)** Tfh frequencies were assessed in the lungs. Total **(K)** and MHC Class I Tetramer NP + **(L)** CD8 T cell frequencies were assessed in the MLN. Total **(M)** and MHC Class I Tetramer NP + **(N)** CD8 T cell frequencies were assessed in the lungs. Statistical significance was calculated by two-way ANOVA with Šídák’s posthoc corrections for pairwise comparisons of control vs. metformin-treated mice. Significance was set at p ≤ 0.05, indicated by *. Trends (0.05 < p ≤ 0.1) indicated by #. **(A,B)**: 6 --8/group, **(C–N)**: 5 --8/group.

We continued evaluating antibody titers, viral load and T cell response at 10 DPSI to determine the effects of various changes with metformin on outcomes to heterologous challenge. When evaluating NP IgG antibodies in the serum and BAL, no significant differences were seen regardless of age or metformin treatment (Figures 4A,B). Lung viral loads were undetectable in the lungs of all cohorts of mice at this time point (all below the lower limit of detection, data not shown). Further, BAL cytokines, e.g., IL-6, TNFa, were not different in metformin treated mice compared to control mice regardless of age (Supplementary Figure S6). Additionally, no differences were observed in total or NP-specific CD4 or CD8 T cells in the MLN at 10 DPSI with metformin treatment (Figures 4C,D). Interestingly, however, aged metformin treated mice had increased NP-specific CD4 T cells, but not total CD4 T cells in the lungs at 10 DPSI (Figures 4D,E). Like earlier times, changes in Tfh were observed in aged mice with treated with metformin. These included a trending increase in NP-specific Tfh CD4 T cell frequency only in the MLN (Figures 4G,H) and a significant increase in total, but not NP-specific Tfh CD4 T cells in the lungs (Figures 4I,J). Metformin treatment did not influence the frequencies of total or NP-specific CD8 T cells in the MLN or lungs at 10 DPSI (Figures 4K–N). Additionally, no significant differences in cell numbers were observed in the MLN and lungs at this timepoint except for a trending increase in NP-specific Tfh CD4 T cells in the MLN in aged metformin treated mice (Supplementary Figure S7). Overall, these results indicate that metformin treatment may alter T cell differentiation and shift towards Tfh cells during a heterologous challenge as we saw increases in total and NP-specific T cells at different timepoints in the MLN and lungs. Interestingly, previous research from our laboratory showed that in healthy older adults, metformin treatment prior to influenza vaccination led to increases in circulating T follicular helper cells (Martin et al., 2023a). Combined, these data suggest that metformin may play a role in promoting Tfh formation in aged immune systems.

### Effect of metformin on resolution of infection responses and inflammation

**FIGURE 5:**
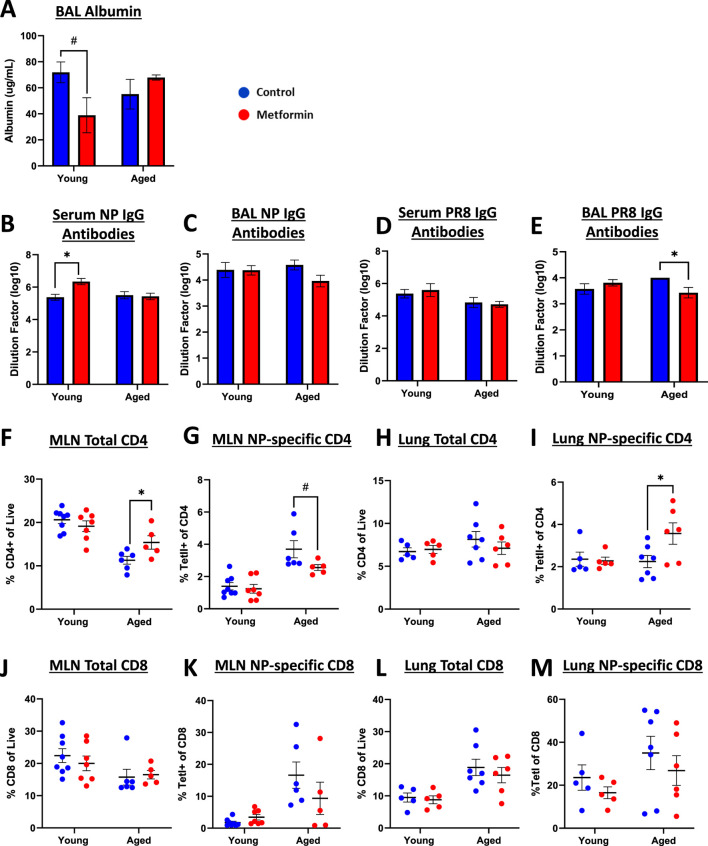
Assessment of immune responses at 14 days post secondary A/Puerto Rico/8/34 (PR8) flu infection. Young and aged, control or metformin treated mice were initially infected with H3N2 influenza virus A/HKx31 (X31), then challenged 30 days later with H1N1 influenza virus A/Puerto Rico/8/34 (PR8) and sacrificed at 14 days post secondary infection. At the time of sacrifice, serum from cardiac puncture, bronchoalveolar lavage (BAL) fluid, the mediastinal lymph node (MLN), and lungs were analyzed. **(A)** BAL Albumin was assessed to measure lung damage. Flu nucleoprotein (NP) IgG antibody levels were assessed in the serum **(B)** and BAL **(C)**. Total PR8 IgG antibody levels were assessed in the serum **(D)** and BAL **(E)**. Total **(F)** and MHC Class II Tetramer NP + **(G)** CD4 T cell frequencies were assessed in the MLN. Total **(H)** and MHC Class II Tetramer NP + **(I)** CD4 T cell frequencies were assessed in the lungs. Total **(J)** and MHC Class I Tetramer NP + **(K)** CD8 T cell frequencies were assessed in the MLN. Total **(L)** and MHC Class I Tetramer NP + **(M)** CD8 T cell frequencies were assessed in the lungs. Significance was set at p ≤ 0.05, indicated by *. Trends (0.05 < p ≤ 0.1) indicated by #. **(A)**: 5/group, **(B–E)**: 6 --8/group, **(F–M)**: 5 --8/group.

Finally, we wanted to evaluate the resolution of infection responses after heterologous challenge. To assess lung damage, we measured albumin concentrations in BAL at 14 DPSI (Figure 5A). Young metformin treated mice had a trending decrease in BAL albumin compared to young control treated mice, but no differences were observed in aged mice. This suggests that metformin may attenuate lung damage during heterologous challenge in young, but not aged mice. Like 10 DPSI, BAL cytokines remained low in all cohorts of mice and showed no differences regardless of age or treatment at 14 DPSI (Supplementary Figure S8). Young metformin treated mice had increased serum NP IgG antibodies, but no difference in BAL NP IgG antibodies compared to young control mice (Figures 5B,C). Aged mice showed no differences in NP IgG antibodies in either the serum or BAL. Whole virus PR8 IgG antibodies were not different in the serum with metformin treatment in young or aged mice (Figure 5D), however aged metformin treated mice had reduced BAL whole virus PR8 IgG antibodies (Figure 5E). It is unclear if this is a sustained change or only a transient effect at this timepoint. Overall, these data suggest that metformin treatment during primary infection may modulate *de novo* responses to PR8 influenza infection such as IgG antibody responses.

Interestingly, aged metformin treated mice had increased frequencies of total CD4 T cells but trending decreased NP-specific CD4 T cell frequency in the MLN compared to control treated aged mice at 14 DPSI (Figures 5F,G). Simultaneously, a decrease in NP-specific CD4 T cell numbers was noted in the MLN of metformin treated aged mice (Supplementary Figure S9). Notably, there was also an increased frequency of NP-specific CD4 T cells, but not total CD4 T cells in the lungs of metformin treated aged mice compared to control treated aged mice (Figures 5H,I), suggesting that the decrease in NP-specific CD4 T cells in the MLN was due to increased trafficking to the lungs. Metformin treatment did not influence the frequencies of total or NP-specific CD8 T cells in the MLN or lungs 14 DPSI (Figures 5J–M). However, a significant decrease in NP-specific CD8 T cell numbers occurred in metformin treated aged mice (Supplementary Figure S9). While metformin treatment alters some aspects of immune responses to secondary flu challenge in both young and aged mice, there are no overt benefits in terms of major flu outcomes, such as flu-induced weight loss and lung viral load. In fact, metformin treated aged mice had increased lung viral load and decreased NP-specific T cells transiently during secondary infection. In totality, our results suggest that metformin treatment does not significantly improve immune responses and memory formation during primary flu infection, resulting in minimal differences in responses to heterologous flu challenge in aged mice. Overall, this highlights that metformin may not be a strong enough stimulus to fully overcome the deficits in T cell memory formation or that alternative mechanisms may come into play with aging to impact metformin’s ability to modulate protective memory responses.

## Discussion

The primary goal of this study was to determine if metformin can be utilized to improve aged T cell memory responses using a heterologous flu challenge model. Metformin, a first line type II diabetes treatment, is a candidate gerotherapeutic. Metformin’s pleotropic effects, especially its ability to reduce chronic inflammation, are of particular importance and have the potential to impact the immune system (Ba et al., 2019; Cameron et al., 2016). Additionally, metformin has been shown to target multiple aging hallmarks including deregulated nutrient sensing, inflammation and altered intracellular communication (Kulkarni et al., 2020). More recently, growing evidence suggests that metformin can be particularly beneficial in targeting age related immune declines (Justice et al., 2021; Martin et al., 2023b). Since immune memory formation is one of the major and most notable declines in immune responses with aging, we sought to determine if metformin could improve aged T cell memory responses. More specifically, we evaluated if metformin could be beneficial in the context of a heterologous flu challenge, as previous research has shown that this drug can promote T cell memory formation in young mice (Pearce et al., 2009). To interrogate this, we pre-treated young and aged mice with metformin or control chow, then administered a primary infection (X31), followed by a secondary infection (PR8). It is important to note that metformin chow was withdrawn at 10 days post primary infection (DPPI) to determine the potential effects of metformin on memory formation, rather than the direct impact of metformin treatment on immune cells during acute infection.

We first evaluated antibody and T cell memory responses to primary X31 flu infection. At 30 DPPI, we observed a trending decrease in BAL NP IgG antibodies and total lung resident memory CD8 T cells in metformin treated aged mice compared to control. Young mice treated with metformin had increased total CD8 and CD4 T cells in the MLN, but not NP-specific populations. This was surprising given previous studies have shown an improvement in CD8 T cell memory responses with metformin in young mice (Pearce et al., 2009). This also suggests that metformin may impact CD4 and CD8 T cells differentially.

Following secondary infection with heterologous PR8 flu, we did not observe any overt benefits with metformin treatment in aged mice, apart from modest improvements in weight loss after 10 DPSI. In fact, we observed a transient increased viral load and inflammatory cytokines including IL-6, TNFα and IL-1β in aged metformin treated mice, however this is resolved by 10 DPSI. At 7 DPSI, a notable inflection point in weight loss also occurred where aged metformin treated mice begin to recover weight while control treated counterparts remain stagnant. This surprising relationship with improved weight recovery, yet elevated BAL inflammatory cytokines and lung viral load is not clear. It is possible that metformin treated mice may have altered tissue repair processes in other tissues that are not directly infected with flu, such as skeletal muscle. This could potentially explain the improved weight recovery during these time points. It is also possible that the increased viral load and BAL inflammation were so transient that other T cell and flu responses were not impacted at all. Additional studies are required to unveil the full effects of this increased inflammation on flu recovery with metformin pre-treatment, especially at intermediate timepoints that were not evaluated in the current study. A notable finding was the consistent increase or trending increase in total and/or NP-Specific T follicular helper (Tfh) CD4 T cells in the MLN and/or lungs of aged metformin treated mice. This suggests that metformin treatment impacts CD4 T cell differentiation patterns in aged mice with specific promotion towards Tfh subsets. Interestingly, previous research from our lab also found that metformin increases circulating Tfh after flu vaccination in healthy, nondiabetic older adults (Martin et al., 2023a). In that clinical trial we did not have sufficient statistical power to detect differences in antibody levels, however here, we also do not observe increased NP IgG antibodies in the serum or BAL of aged mice. This suggests that while metformin may promote differentiation towards Tfh, it may not improve Tfh functionality, like the ability to provide B cell help, to the level required to improve aged B cell responses and antibody production. Surprisingly, while we did not observe differences in Tfh in young metformin treated mice, we did observe higher levels of NP IgG antibodies in BAL at 5 DPSI and serum NP IgG antibodies at 14 DPSI in young mice. It is possible that metformin may have had more impact on young B cell responses, however this was outside the scope of the current project. This would align, however, with previous research demonstrating metformin’s ability to boosts B cell function in response to the seasonal flu vaccine in both young and older individuals with type 2 diabetes (Frasca et al., 2021; Diaz et al., 2017). Nonetheless, the similarities of increased Tfh in both clinical trials and mouse studies point to underlying mechanisms of metformin to promote Tfh skewing that should be explored further. Future studies are required to understand how metformin impacts CD4 T cell differentiation towards Tfh and the meaningfulness of increased Tfh in these contexts.

Although our study was designed to interrogate age related changes and potential benefits of metformin on immune responses and memory formation after flu challenge, some interesting changes occurred in young mice. For example, during secondary infection, young mice showed a significant increase in BAL NP IgG antibodies at 5 DPSI, suggesting that metformin improved recall responses to NP, as mice also respond to NP as a part of primary infection due to its conserved nature. Interestingly, this increase in antibody titers was not associated with detectable changes in Tfh responses. This suggests that metformin may influence other crucial components of humoral immunity such as B cell activation, recruitment or the quality of T-B cell interactions. It is also possible that our evaluated timepoints were not sufficient in capturing the dynamic changes in T cells responses, and that a detectable difference occurred at a non-evaluated time points, resulting in this increase in antibodies. Lastly, some interesting changes were observed in metformin treated young mice at 14 DPI including a trending decrease in BAL albumin, a measure of lung damage, along with a significant increase in serum NP IgG antibodies. In combination with the increase of BAL NP IgG antibodies in these mice at 5 DPSI, it is possible that metformin treatment in young mice potentiates NP IgG antibody responses but does not impact *de novo* antibody responses during secondary infection. Unlike in aged mice, however, young mice do not have a clear difference in Tfh CD4 T cell responses, but rather an impact only on total cell populations. Additional interrogation of the effects of metformin on CD4 T cell subsets is required; however, this was not the focus of this current study. Furthermore, additional studies are required to better understand the effects and magnitude of metformin treatment on immunometabolic regulation, specifically examining the impact on AMPK/mTOR signaling pathways and other downstream signaling. These studies may unveil key differences in why young mice showed changes, while only having some modest changes in aged mice in our study. This information may also inform future studies of the metformin dosage requirement and/or duration of treatment needed to induce significant, long term immunomodulatory changes in aged male mice, however, was outside the scope of the current study. This study may also serve as important preliminary guidance for future studies focused on female mice as various aspects of immune aging and infection, as well as age-related changes in metabolism have been shown to differ in females compared to males.

One limitation of this study was that we focused our analyses on NP-specific T cells and their subsets to determine whether metformin could improve T cell memory formation. Although we examined other metrics such as serum and BAL antibody titers, lung viral load, and lung damage, more research is necessary to determine if metformin impacts other immune cell populations, such as including innate immune cells, other antigen-specific T cell subsets and B cell populations. Previous research has shown that metformin improves B cell function and antibody responses in older adults with type II diabetes (Frasca et al., 2021; Diaz et al., 2017). While our lack of changes in antibody titers in aged mice suggest that B cells responses were not significantly impacted by metformin treatment, further research would be beneficial in determining if metformin can modulate B cells responses or memory formation in other contexts. Continued studies interrogating T cell subsets such as naïve, central memory and effector memory cells would be beneficial in further evaluating the effects of metformin in the context of aging and infection. This evaluation would provide important insights on the potential robustness of immune responses compared to the functional protection generated. An additional limitation of our study stems from our experimental timeline. Here, we discontinued metformin treatment 10 days after primary flu challenge to specifically assess its effects on immune memory formation. However, the outcomes of this study may have differed if metformin treatment had been extended throughout primary or secondary infection. Interestingly, previous studies have shown that obese individuals with a history of metformin use exhibit lower influenza mortality (Cummings et al., 2022) and infection rates (Mor et al., 2016), suggesting prolonged treatment may exert stronger or distinct effects compared to those observed in the current study.

Although a case has been made that metformin can be repurposed to target age related deficits, and particularly immune declines with aging (Justice et al., 2021; Martin et al., 2023b). Our study does not show overt benefits of metformin in the context of memory T cell formation and protective responses to secondary flu challenge in aged mice. This is particularly surprising given the previously shown benefits of metformin treatment in T cell memory formation in young mice (Pearce et al., 2009). Thus, it is possible that the dose metformin used in our studies did not provide a strong enough stimulus to activate AMPK and promote T cell memory responses. Future mechanistic studies are required to confirm the exact effect of metformin on AMPK/mTOR and other immunometabolic pathways. These would provide crucial insights into the utility of metformin in aging, infection and other disease states. Our studies used 0.1% metformin treated chow, like the NIA Intervention Testing Program (ITP) and other studies showing benefits of metformin in healthspan and lifespan in mice (Martin-Montalvo et al., 2013). Additionally, metformin alone may not be sufficient to overcome the totality of aging immune deficits. More research is necessary to further elucidate the mechanisms of metformin and determine its specific potential to improve different aspects of age-related immune declines and its effects across various disease states.

## Conclusion

Effective protection against infectious disease and associated complications is of upmost importance for aging populations. This study investigated if metformin, an FDA approved diabetes drug, could be repurposed to improve age-related declines in T cell memory responses in the context of a heterologous flu challenge. Although some differences in T cell responses were observed in aged mice treated with metformin, including some instances of increased CD4 T cells and CD8 T cells in the MLN and lungs, metformin did not significantly improve overall flu outcomes. Young mice treated with metformin have improved antibody responses and reduced lung damage after secondary infection; however this was not observed in aged mice. Further research is needed to determine if metformin may have utility in addressing age-related immune declines more broadly at higher doses, with prolonged treatment and/or in the context of vaccination responses. Understanding the mechanism of metformin’s impact on immune responses with aging is essential to develop appropriate treatment and prevention methods for infection-induced pathologies.

## Data Availability

The raw data supporting the conclusions of this article will be made available by the authors, without undue reservation.

## Supplementary material

SUPPLEMENTARY TABLE S1 Metformin diet formulation.

SUPPLEMENTARY TABLE S2 Antibody details used for flow cytometry analysis.

SUPPLEMENTARY FIGURE S1 Assessment of BAL cytokines at Day 30 post primary A/HKx31 (X31) flu infection. Young and aged, control or metformin treated mice were initially infected with H3N2 influenza virus A/HKx31 (X31), then challenged 30 days later with H1N1 influenza virus A/Puerto Rico/8/34 (PR8) and sacrificed at day 0. The bronchoalveolar lavage fluid (BAL) fluid was collected and analyzed for cytokine concentrations via multiplex. Statistical significance was calculated by two-way ANOVA with Šídák’s posthoc corrections for pairwise comparisons of control vs. metformin-treated mice. Significance was set at p ≤ 0.05, indicated by *. Trends (p ≤ 0.1) indicated by #. Trends (0.05 < p ≤ 0.1) indicated by #. Significant main age effects were seen for GCSF. n = 4–5/group.

SUPPLEMENTARY FIGURE S2 Assessment of baseline immune responses at 30 days post primary A/HKx31 (X31) flu infection. Young and aged, control or metformin treated mice were infected with H3N2 influenza virus A/HKx31 (X31). Mice were sacrificed at day 0 and mediastinal lymph node (MLN), and lungs were analyzed via flow cytometry. A- D) Total and MHC Class II Tetramer NP + CD4 T cell numbers were assessed in the MLN and lungs. E − H) Total and MHC Class I Tetramer NP + CD8 T cell numbers were assessed in the MLN and lungs. I - L) Total and MHC Class I and II tetramer NP + resident memory (CD69^+^CD103+) CD4 and CD8 T cells were assessed in the lungs. Statistical significance was calculated by two-way ANOVA with Šídák’s posthoc corrections for pairwise comparisons of control vs. metformin-treated mice. Significance was set at p ≤ 0.05, indicated by *. Trends (p ≤ 0.1) indicated by #. Trends (0.05 < p ≤ 0.1) indicated by #. Significant main age effects were seen for lung total CD4, lung flu-specific CD4, lung total CD8, lung flu-specific CD8, and total and flu-specific resident memory CD4 and CD8 T cells. N = 5–7/group.

SUPPLEMENTARY FIGURE S3 Assessment of immune cell counts at Day 5 post secondary A/Puerto Rico/8/34 (PR8) flu infection. Young and aged, control or metformin treated mice were infected with H3N2 influenza virus A/HKx31 (X31), then challenged 30 days later with H1N1 influenza virus A/Puerto Rico/8/34 (PR8). Mice were sacrificed at 5 days post secondary infection and mediastinal lymph node (MLN), and lungs were analyzed via flow cytometry. A- D) Cell counts of total and MHC Class II Tetramer NP + CD4 T cell numbers were evaluated in the MLN and lung. E − H) Total and MHC Class II Tetramer NP + T follicular helper (Tfh; BCL6+FOXP3-) CD4 T cell numbers were assessed in the MLN and lungs. I - L) Total and MHC Class I Tetramer NP + CD8 T cell numbers were assessed in the MLN and lungs. Statistical significance was calculated by two-way ANOVA with Šídák’s posthoc corrections for pairwise comparisons of control vs. metformin-treated mice. Significance was set at p ≤ 0.05, indicated by *. Trends (0.05 < p ≤ 0.1) indicated by #. Significant main age effects were seen for lung total CD4, lung flu-specific CD4, lung Tfh, lung total CD8, and lung flu-specific CD8. N = 4–5/group.

SUPPLEMENTARY FIGURE S4 Assessment of BAL cytokines at Day 7 days post secondary A/Puerto Rico/8/34 (PR8) flu infection. Young and aged, control or metformin treated mice were infected with H3N2 influenza virus A/HKx31 (X31), then challenged 30 days later with H1N1 influenza virus A/Puerto Rico/8/34 (PR8) and sacrificed at 7 days post secondary infection. The bronchoalveolar lavage fluid (BAL) fluid was collected and analyzed for cytokine concentrations via multiplex. Statistical significance was calculated by two-way ANOVA with Šídák’s posthoc corrections for pairwise comparisons of control vs. metformin-treated mice. Significance was set at p ≤ 0.05, indicated by *. Trends (0.05 < p ≤ 0.1) indicated by #. Significant main age effects were seen for GCSF, IL-1a, IL-1b, IFN-y, KC, IP-10 (CXCL10), TNFa, IL-12, IL-6, IL-4, and IL-10. N = 4–5/group.

SUPPLEMENTARY FIGURE S5 Assessment of immune cell counts at Day 7 post secondary A/Puerto Rico/8/34 (PR8) flu infection. Young and aged, control or metformin treated mice were infected with H3N2 influenza virus A/HKx31 (X31), then challenged 30 days later with H1N1 influenza virus A/Puerto Rico/8/34 (PR8). Mice were sacrificed at 7 days post secondary infection and mediastinal lymph node (MLN), and lungs were analyzed via flow cytometry. A- D) Total and MHC Class II Tetramer NP + CD4 T cell numbers were evaluated in the MLN and lungs. E − H) Total and MHC Class I Tetramer NP + CD8 T cell numbers were assessed in the MLN and lungs. Statistical significance was calculated by two-way ANOVA with Šídák’s posthoc corrections for pairwise comparisons of control vs. metformin-treated mice. Significance was set at p ≤ 0.05, indicated by *. Trends (p ≤ 0.1) indicated by #. Significant main age effects were seen for MLN flu-specific CD4 T cells. N = 4–7/group.

SUPPLEMENTARY FIGURE S6 Assessment of BAL cytokines at Day 10 post secondary A/Puerto Rico/8/34 (PR8) flu infection. Young and aged, control or metformin treated mice were infected with H3N2 influenza virus A/HKx31 (X31), then challenged 30 days later with H1N1 influenza virus A/Puerto Rico/8/34 (PR8) and sacrificed 10 days post secondary infection. A–J) Bronchoalveolar lavage fluid (BAL) fluid was collected and analyzed for cytokine concentrations via multiplex. Statistical significance was calculated by two-way ANOVA with Šídák’s posthoc corrections for pairwise comparisons of control vs. metformin-treated mice. Significance was set at p ≤ 0.05, indicated by *. Trends (0.05 < p ≤ 0.1) indicated by #. Significant main age effects were seen for KC. Trending main age effects were seen for G-CSF, IL-1a, TNFa, and IL-6. N = 5–7/group.

SUPPLEMENTARY FIGURE S7 Assessment of immune cell counts at Day 10 post secondary A/Puerto Rico/8/34 (PR8) flu infection. Young and aged, control or metformin treated mice were infected with H3N2 influenza virus A/HKx31 (X31), then challenged 30 days later with H1N1 influenza virus A/Puerto Rico/8/34 (PR8). Mice were sacrificed 10 days post secondary infection and mediastinal lymph node (MLN), and lungs were analyzed via flow cytometry. A- D) Total and MHC Class II Tetramer NP + CD4 T Cell numbers were evaluated in the MLN and lungs. E − H) Total and MHC Class II Tetramer NP + T follicular helper (Tfh; BCL6+FOXP3-) CD4 T cell numbers were assessed in the MLN and lung. I - L) Total and MHC Class I Tetramer NP + CD8 T cell numbers were assessed in the MLN and lungs. Statistical significance was calculated by two-way ANOVA with Šídák’s posthoc corrections for pairwise comparisons of control vs. metformin-treated mice. Significance was set at p ≤ 0.05, indicated by *. Trends (0.05 < p ≤ 0.1) indicated by #. Significant main age effects were seen for lung total CD4, lung flu-specific CD4, lung Tfh, lung flu-specific Tfh, lung total CD8, and lung flu-specific CD8. N = 5–8/group.

SUPPLEMENTARY FIGURE S8 Assessment of BAL cytokines at Day 14 post secondary A/Puerto Rico/8/34 (PR8) flu infection. Young and aged, control or metformin treated mice were infected with H3N2 influenza virus A/HKx31 (X31), then challenged 30 days later with H1N1 influenza virus A/Puerto Rico/8/34 (PR8) and sacrificed 14 days post secondary infection. The bronchoalveolar lavage fluid (BAL) fluid was collected and analyzed for cytokine concentrations via multiplex. Statistical significance was calculated by two-way ANOVA with Šídák’s posthoc corrections for pairwise comparisons of control vs. metformin-treated mice. Significance was set at p ≤ 0.05, indicated by *. Trends (0.05 < p ≤ 0.1) indicated by #. N = 4–5/group.

SUPPLEMENTARY FIGURE S9 Assessment of immune cell counts at Day 14 post secondary A/Puerto Rico/8/34 (PR8) flu infection. Young and aged, control or metformin treated mice were infected with H3N2 influenza virus A/HKx31 (X31), then challenged 30 days later with H1N1 influenza virus A/Puerto Rico/8/34 (PR8). Mice were sacrificed 14 days post secondary infection and mediastinal lymph node (MLN), and lungs were analyzed via flow cytometry. A- D) Total and MHC Class II Tetramer NP + CD4 T cell numbers were evaluated in the MLN and lungs. E − H) Total and MHC Class I Tetramer NP + CD8 T cell numbers were assessed in the MLN and lungs. Statistical significance was calculated by two-way ANOVA with Šídák’s posthoc corrections for pairwise comparisons of control vs. metformin-treated mice. Significance was set at p ≤ 0.05, indicated by *. Trends (0.05 < p ≤ 0.1) indicated by #. Significant main age effects were seen for lung total CD4, lung flu-specific CD4, MLN flu-specific CD4, lung total CD8, and lung flu-specific CD8. Aging effect Lung NP + CD8 p = 0.0017. N = 5–8/group.
