# Supplementary figures and images for: The effect of metformin treatment during primary influenza infection on heterologous challenge in young and aged mice
Source: Front Aging. 2026 Jun 17;7:1736105. doi: 10.3389/fragi.2026.1736105 (PMC13318952; doi:10.3389/fragi.2026.1736105)

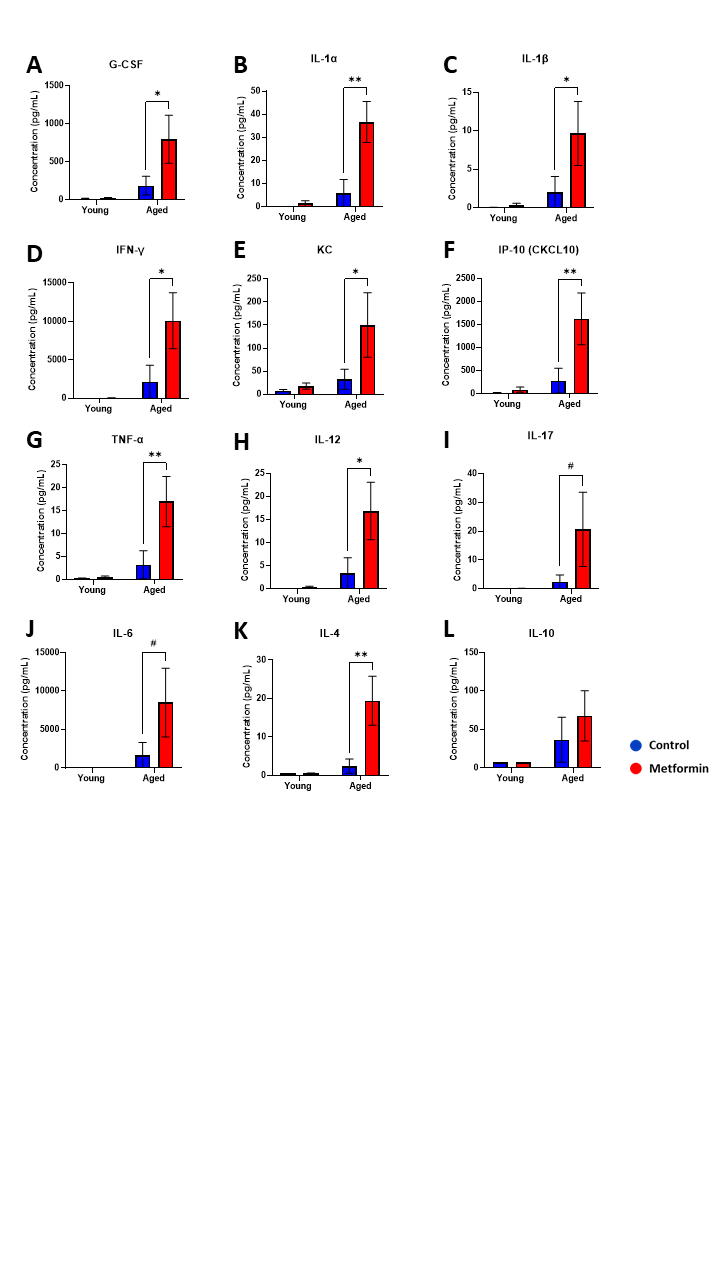

Supplement: Supplementary file 2 [file Image6.tif]

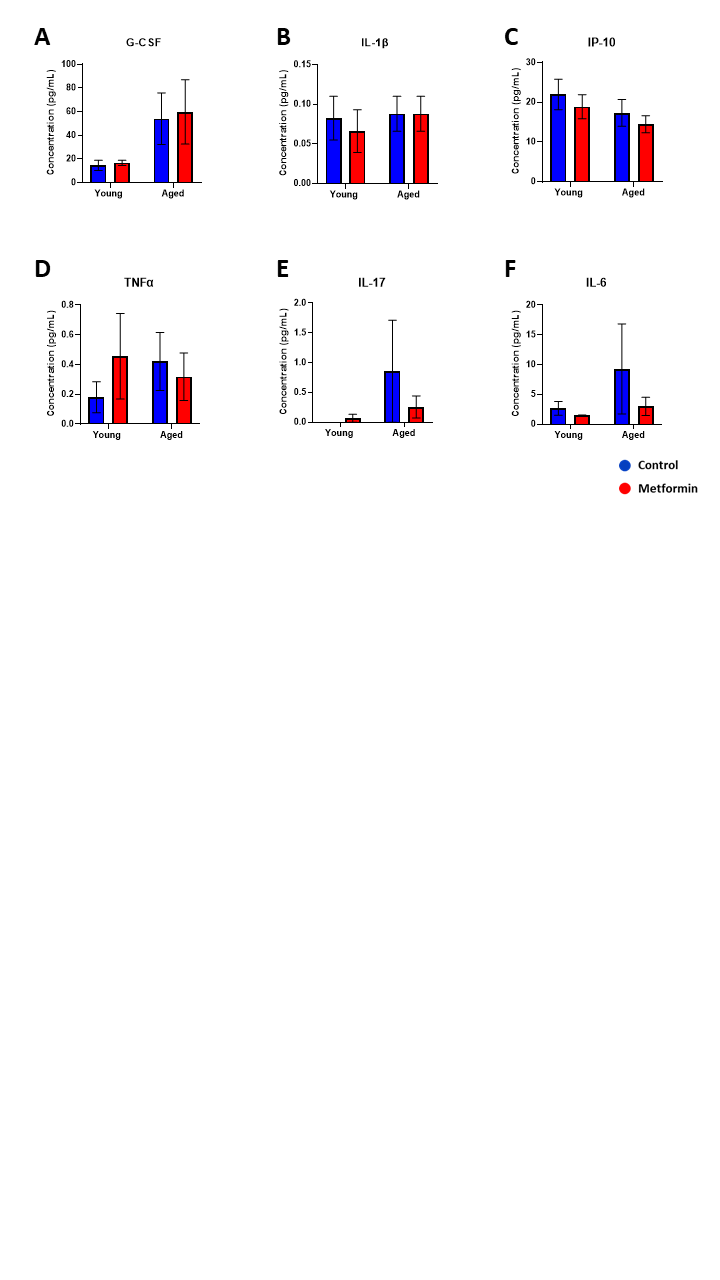

Supplement: Supplementary file 3 [file Image3.tif]

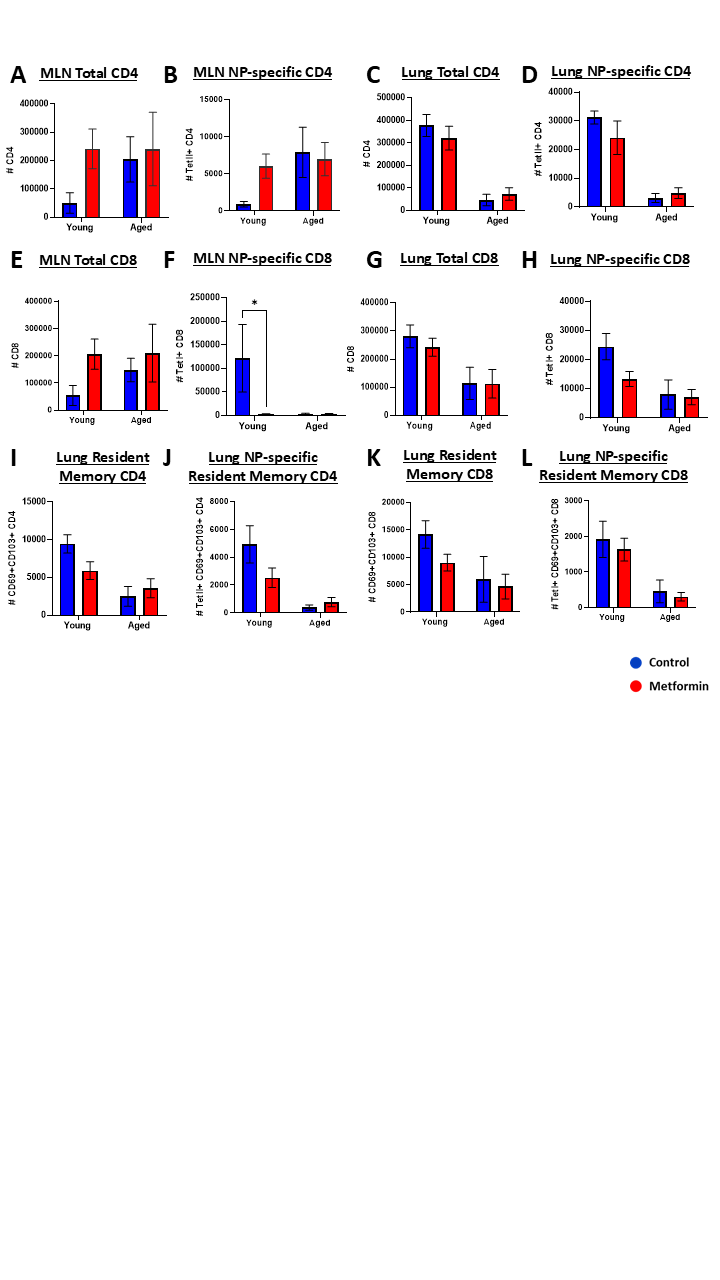

Supplement: Supplementary file 4 [file Image4.tif]

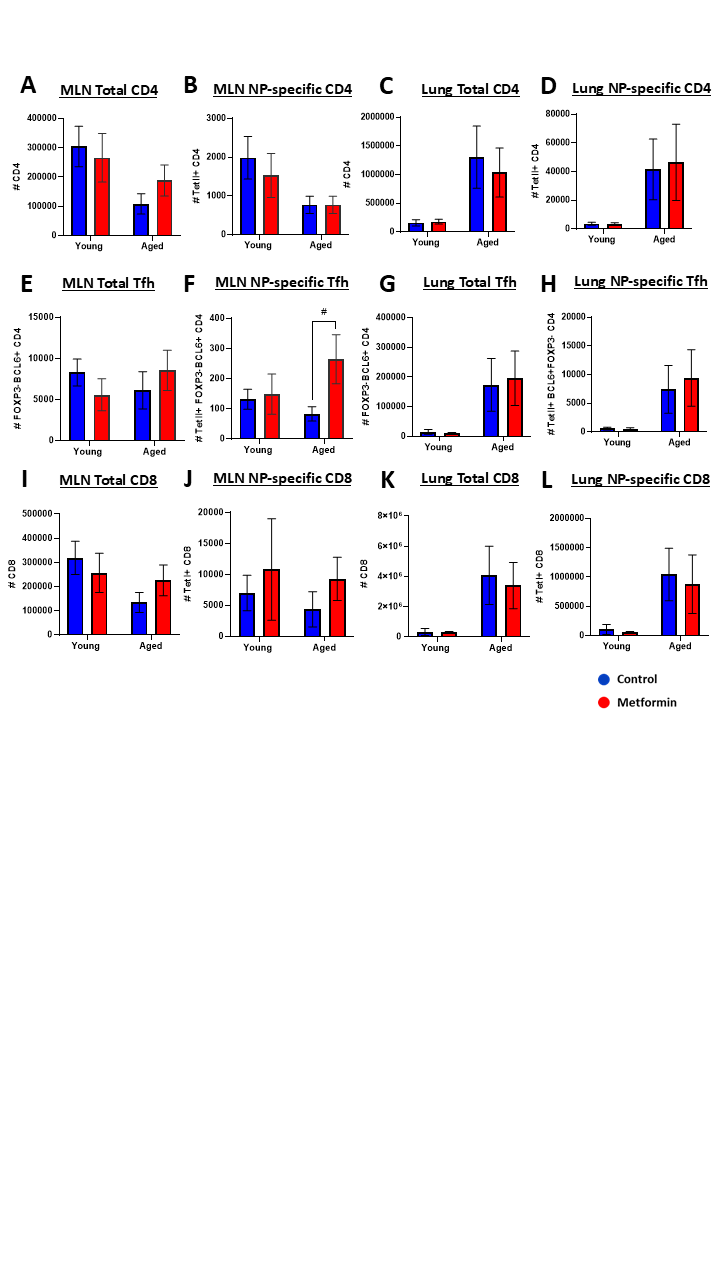

Supplement: Supplementary file 5 [file Image9.tif]

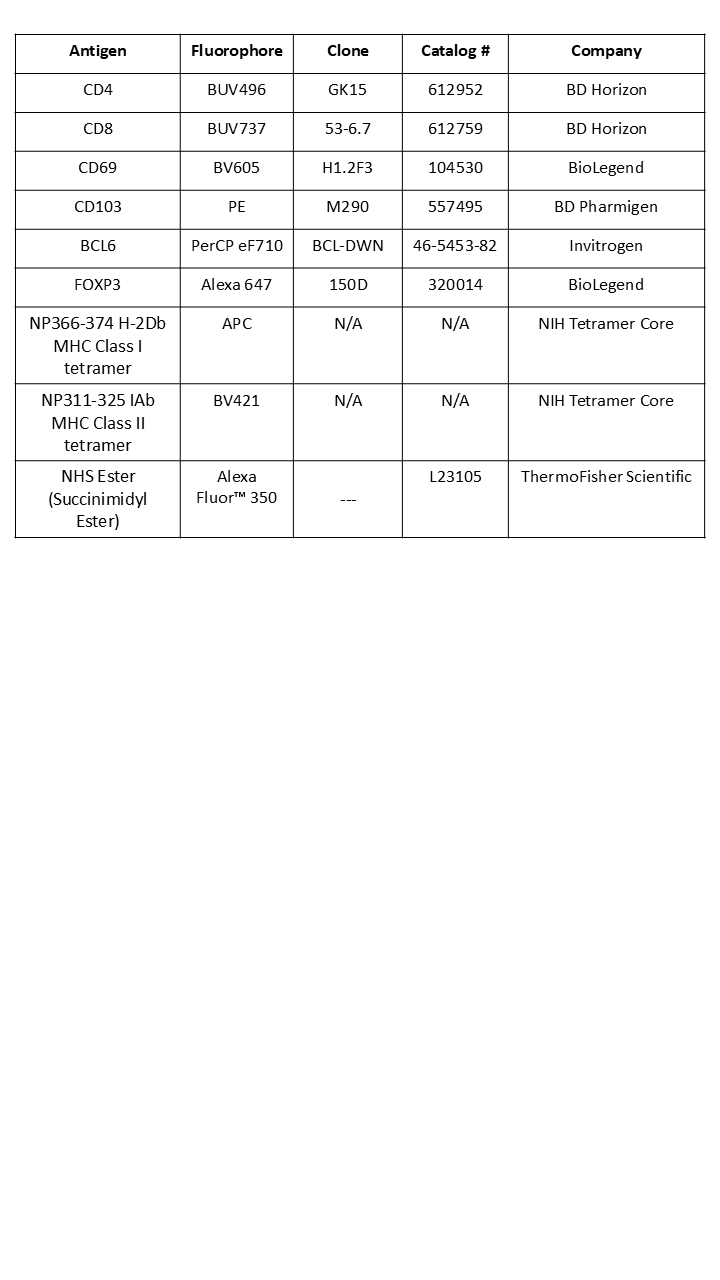

Supplement: Supplementary file 6 [file Image2.tif]

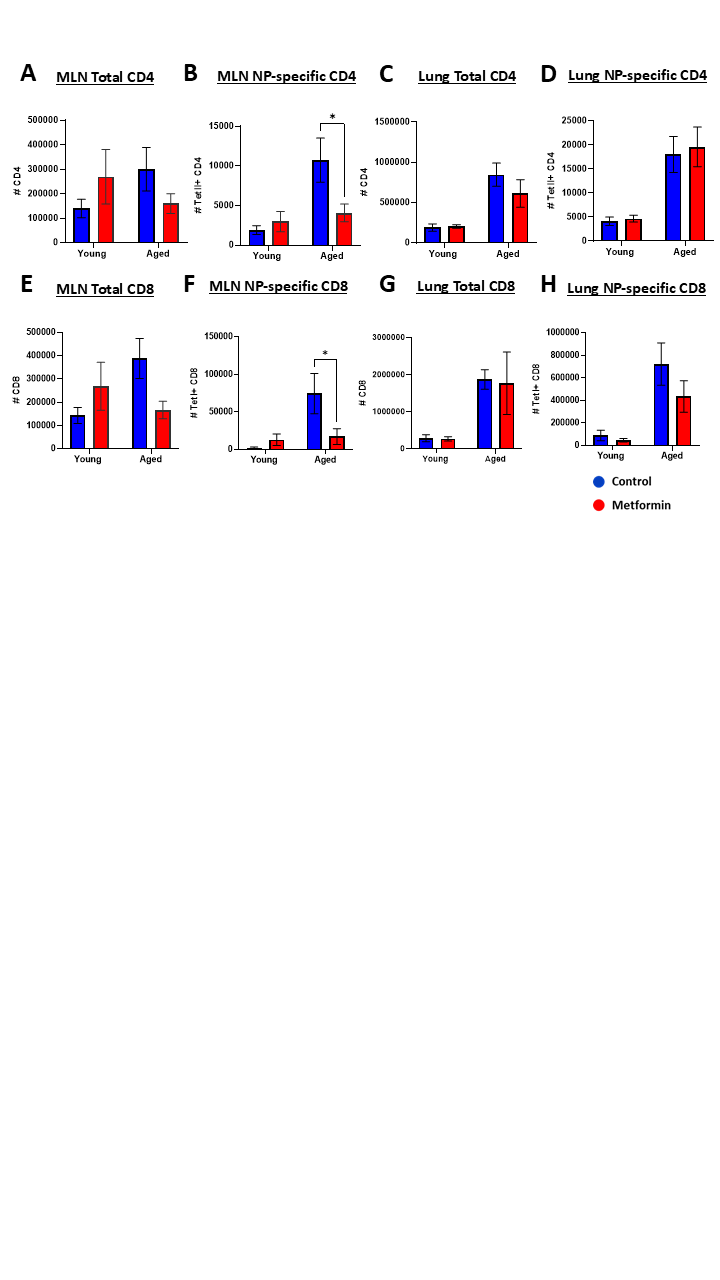

Supplement: Supplementary file 7 [file Image11.tif]

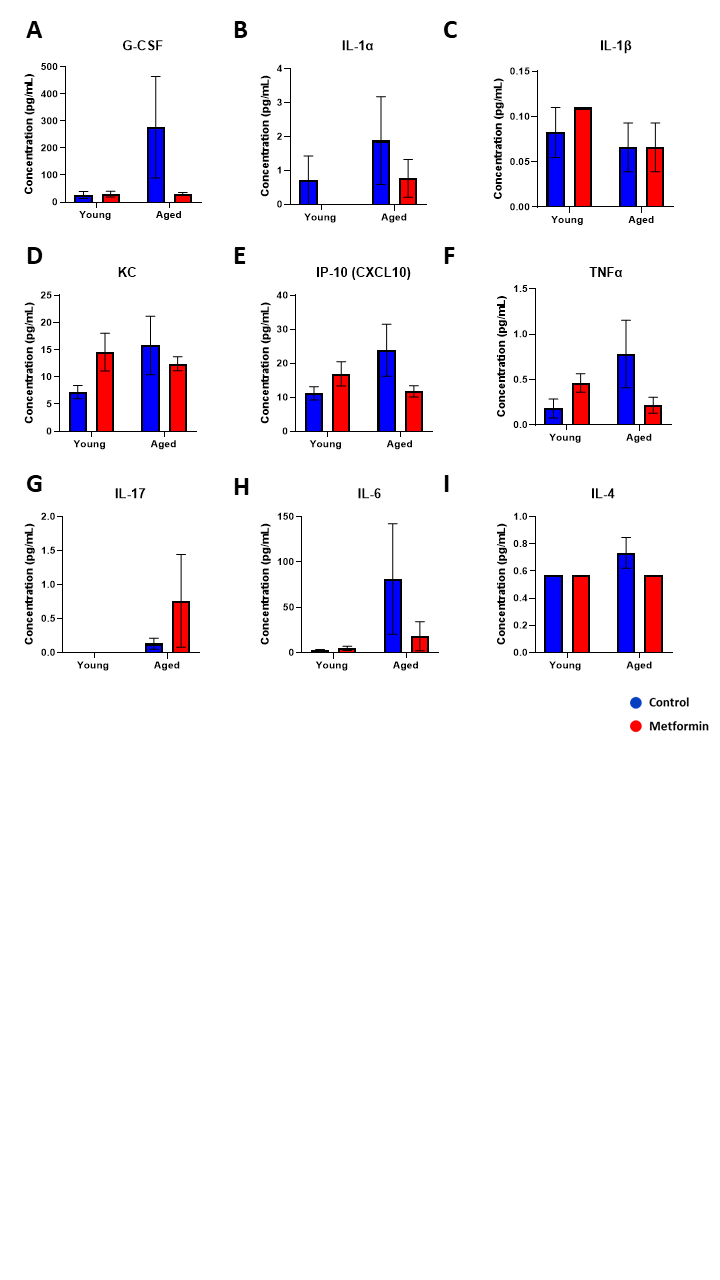

Supplement: Supplementary file 8 [file Image10.tif]

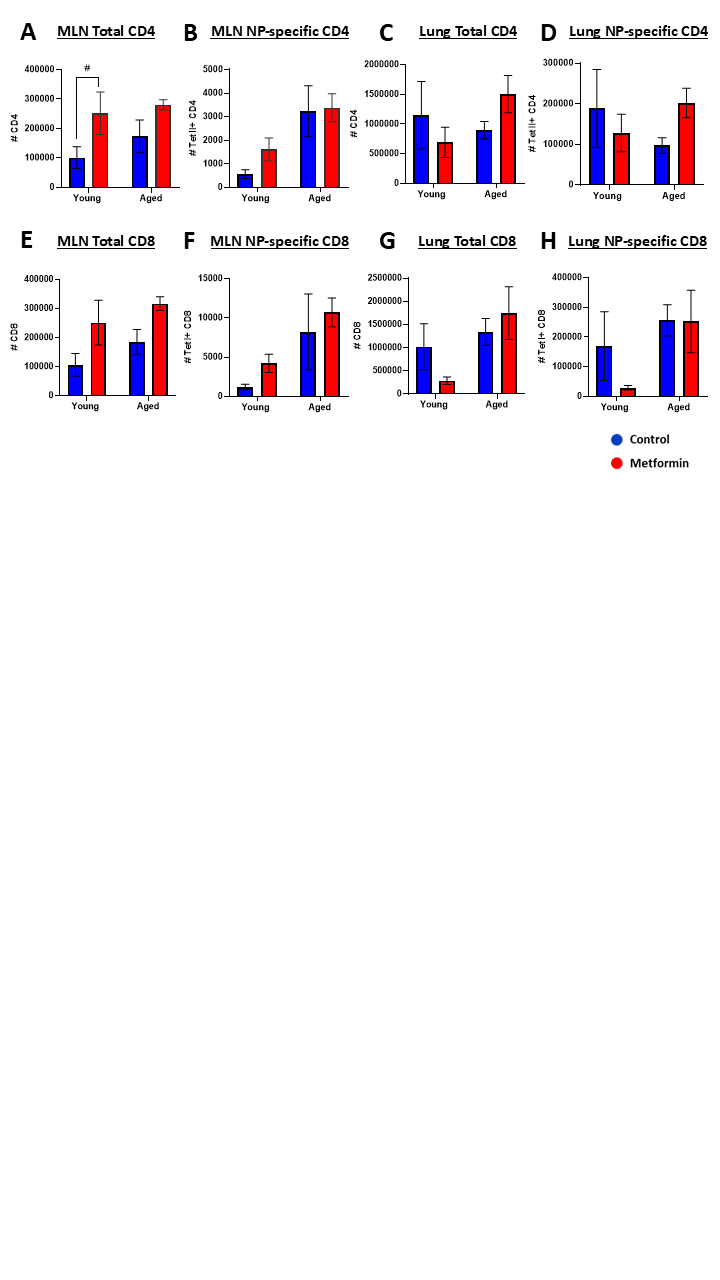

Supplement: Supplementary file 9 [file Image7.tif]

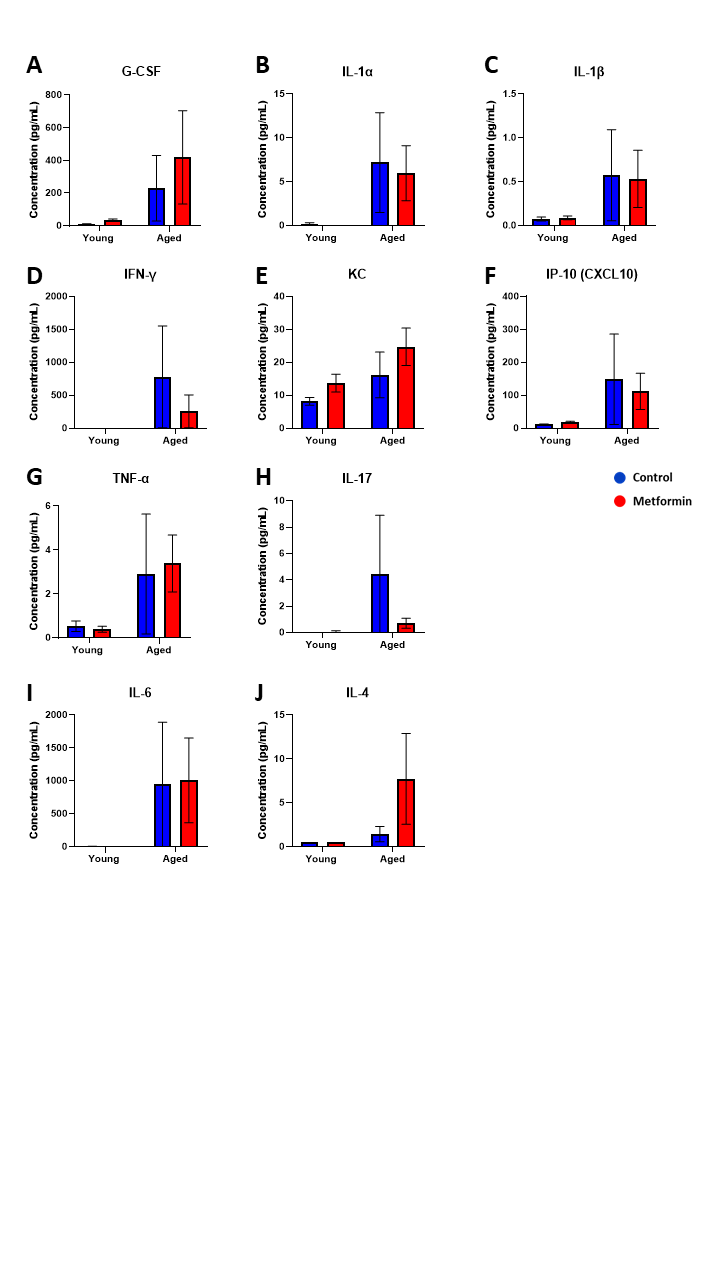

Supplement: Supplementary file 10 [file Image8.tif]

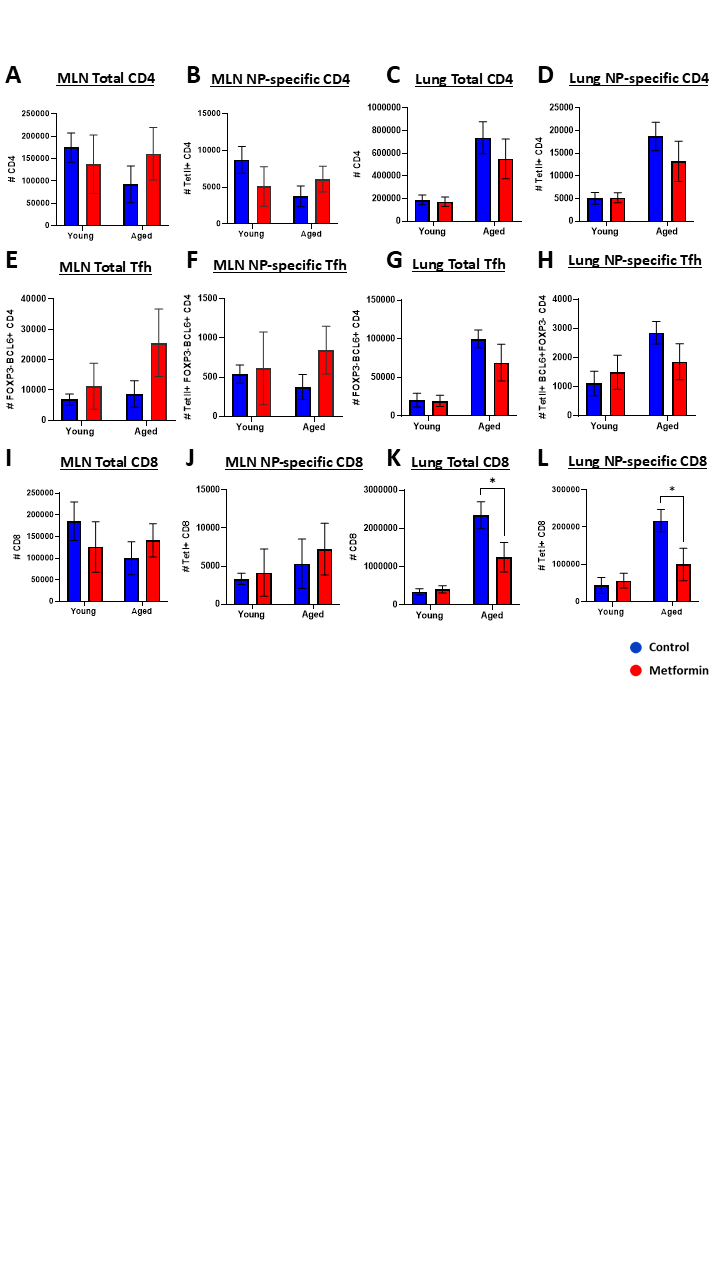

Supplement: Supplementary file 11 [file Image5.tif]
